# Determinants of Access to Sexual and Reproductive Health for Adolescent Girls in Vulnerable Situations in Latin America

**DOI:** 10.3390/ijerph22020248

**Published:** 2025-02-10

**Authors:** Fabian Dávila, Favio Cala-Vitery, Luz-Tatiana Gómez

**Affiliations:** Public Policy Modeling and Management, School of Natural Sciences and Engineering, Universidad de Bogotá Jorge Tadeo Lozano, Bogotá 111711, Colombia; favio.cala@utadeo.edu.co (F.C.-V.); luzt.gomez@utadeo.edu.co (L.-T.G.)

**Keywords:** sexual and reproductive health, adolescents, barriers and facilitators, Latin America, intersectoral collaboration, public policy, social stigma, structural inequalities

## Abstract

Latin American upper-middle-income countries (LAUMICs) face significant challenges in ensuring equitable access to sexual and reproductive health (SRH) for adolescents due to socioeconomic inequalities, migration, and historical violence. This systematic review examines the barriers and facilitators to sexual and reproductive health access for adolescent girls (≤19 years old) in vulnerable situations across Latin America. Using six databases, 391 articles were reviewed; 10 met the inclusion criteria, and 10 more were included through backward search, resulting in 20 analyzed studies. Structural barriers such as economic, regulatory, and institutional constraints were found to restrict SRH access, exacerbated by social stigma and negative narratives, especially in cases involving sexual violence and clandestine abortions. Facilitators included intersectoral collaboration, preventive education, and community networks, which improved access to and the quality of services for vulnerable populations. The findings emphasize the need for sustainable strategies that address structural inequalities and stigma while strengthening health systems, and intersectoral cooperation. Comprehensive, culturally tailored education and clear regulatory frameworks are essential to achieving equitable and sustainable SRH services. These insights offer practical guidance for public policy and program design in LAUMICs, particularly for addressing the needs of marginalized adolescent populations.

## 1. Introduction

The sexual and reproductive health (SRH) of adolescent girls is vital to their overall well-being and development, particularly in vulnerable contexts. However, access to essential SRH information and services, as well as the ability to exercise sexual and reproductive rights equitably and autonomously, is frequently obstructed by socio-cultural barriers. These include entrenched gender roles, stigma surrounding adolescent sexuality, and cultural norms that discourage access to SRH services, especially for marginalized groups. These barriers are further compounded by regional disparities and the limited capacity of health systems.

Adolescence is defined as the age range of 10–19 years, in accordance with the World Health Organization (WHO) guidelines. Adolescents from ethnic minorities, displaced populations, and migrant communities are disproportionately affected by these challenges, which amplify their vulnerabilities and perpetuate existing inequities s, particularly for marginalized populations.

Social stigma and cultural factors amplify these challenges, especially in communities where entrenched gender norms dictate limited autonomy for adolescent girls. For instance, unmarried pregnant adolescents often face ostracization, while ethnic minorities may experience discrimination [1,2,3,4,5,6,7,8].

Early pregnancy—defined as occurring before the age of 20—is a prominent example of the adverse outcomes resulting from inadequate SRH access. Beyond elevated obstetric and perinatal risks, early pregnancy has long-term implications for adolescent girls, including disrupted education, reduced economic opportunities, and broader societal impacts, such as diminished human capital and social cohesion [9].

Economically, countries in Latin America such as Argentina, Brazil, Colombia, Costa Rica, and Mexico, among others are classified as upper-middle-income countries (UMICs) by the World Bank. This classification signifies certain economic advancements, such as increased average incomes, but fails to address persistent inequalities affecting vulnerable populations. For countries previously designated as low or middle income, the UMIC classification often restricts access to international funding, as global priorities shift toward lower-income economies. This shift forces local governments to rely heavily on domestic policies and resources, which are often inadequate or ineffective in meeting the needs of marginalized groups [10,11,12,13,14,15,16,17,18].

## 2. Materials and Methods

This study utilized a systematic review design to identify barriers and facilitators to sexual and reproductive health (SRH) access for adolescent women (≤19 years) residing in lower-middle-income countries in Latin America (LAUMICs), focusing on contexts involving ethnic and racial minorities, migration, and forced displacement.

Search Strategy: The search was conducted across six indexed databases: PubMed, Embase, Scopus, Lilacs, Dimensions, and Google Scholar. Following the guidelines of the JBI Manual for Evidence Synthesis, the PIC framework (Population, Phenomenon of Interest, and Context) guided the strategy (see Table A1).

- Population: Adolescent women (≤19 years) living in LAUMICs.
- Phenomenon of Interest: Barriers and facilitators for access to SRH, including sex education, contraception, and termination of pregnancy.
- Context: Ethnic and racial minorities, migration, and forced displacement.

The primary research question was as follows: What barriers and facilitators for SRH access are identified among adolescent women (≤19 years) in LAUMICs who belong to ethnic minorities, are migrants, or face forced displacement?

Selection and Screening: Eligible studies included observational, qualitative, and mixed-method designs. Additional references were identified through backward citation searches. The inclusion criteria are outlined in Table A2, and reference management was conducted using Rayyan AI (web-based version, updated 30 April 2024).

Rayyan AI was employed to streamline the screening process by facilitating the identification of duplicates and enabling blinded, independent review by two researchers. This tool significantly improved efficiency and consistency during the study’s selection process.

The selection process included three stages:

1. Duplicate Removal: Duplicates were identified and removed using automated Rayyan functions and manual review.
2. Primary Screening: Two reviewers evaluated titles and abstracts independently under a blinded procedure. Conflicts were resolved by consensus.
3. Secondary Screening: Full texts of selected articles were reviewed, and additional relevant documents were identified through string searches [19].

The screening process and results were summarized using a PRISMA-compliant flow chart [20].

Quality Assessment and Data Extraction: Two reviewers independently assessed study eligibility and quality using the JBI Critical Appraisal Checklist for Qualitative Research [21]. The JBI criteria for qualitative research were also applied to mixed-methods studies, as their qualitative components were crucial for addressing the research question and offered the most relevant insights. Any discrepancies in assessment were resolved through discussion to ensure consistency and accuracy.

Quality scores were assigned as follows:

- High quality: ≥8/10 positive responses (>70%).
- Medium quality: 5–7/10 positive responses (50–70%).
- Low quality: <5/10 positive responses (<50%).

Metadata from selected studies were exported from Rayyan.ai into Microsoft Excel. Additional fields not captured by Rayyan, such as study methodology, geographic location, participant characteristics, and conclusions, were extracted as per QARI guidelines [22].

Qualitative Synthesis: A meta-aggregation approach was used to synthesize findings at three levels:

4. Individual Findings: Barriers and facilitators identified in each study.
5. Categories: Grouping findings by similarity.
6. Synthesized Findings: Generalizable statements to inform practice and policy.

All methods and protocols adhere to guidelines for systematic reviews and are detailed to ensure reproducibility. Ethical considerations were not applicable as no new data collection involving human or animal subjects was conducted.

## 3. Results

### 3.1. Search and Selection Strategy

**Figure 1:**
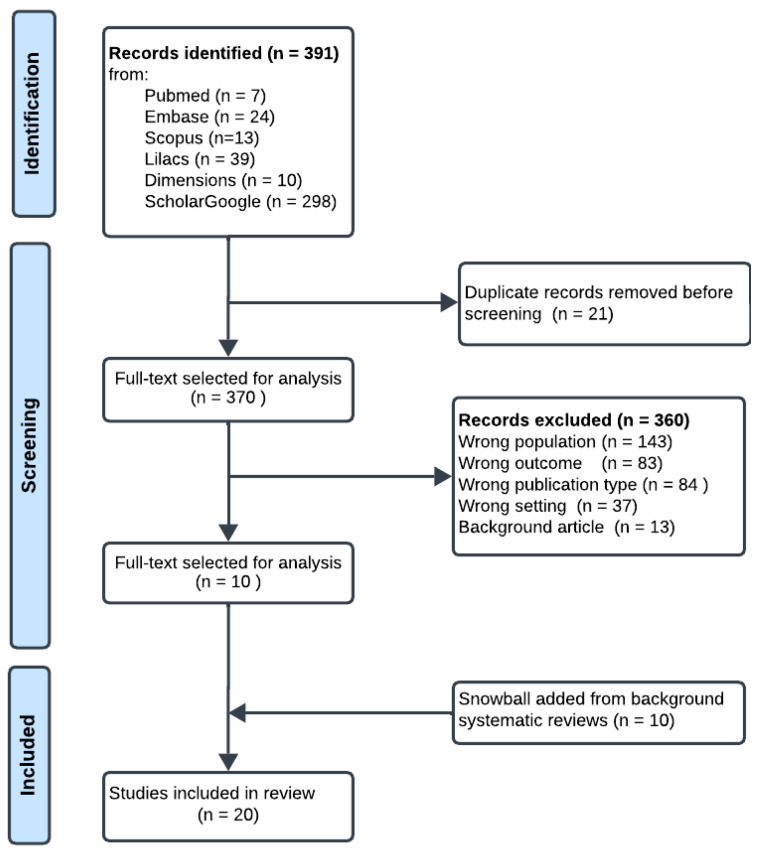
Identification of studies via databases. Adapted from the PRISMA 2020 statement: an updated guideline for reporting systematic reviews [20].

Of the 391 articles initially identified, 21 duplicates were removed. The remaining articles were screened for relevance, leading to the exclusion of 360 records. Detailed reasons for exclusion include the following: wrong population (*n* = 143), referring to studies focused on groups outside the target population; wrong outcome (*n* = 83), referring to studies with endpoints unrelated to the research question; wrong publication type (*n* = 84), such as editorials, expert opinions, or other non-research formats; wrong setting (*n* = 37), referring to studies conducted outside the scope of Latin American upper-middle-income countries (LAUMCs); and background articles (*n* = 13), which provided general information without directly addressing the study objectives (see Table A2). An additional 10 studies were included through snowball sampling from background systematic reviews. This process resulted in a final selection of 20 studies for analysis (see Figure 1 and Table A4).

### 3.2. Critical Appraisal

Table 1 presents the critical appraisal of the 20 included studies. The majority (16 studies) were evaluated as high quality, while four were rated as moderate. Common deficiencies included insufficient clarity on the researcher’s cultural or theoretical positioning (Q6) and inadequate consideration of the investigator’s influence on the research process (Q7).

Key Improvement Area: Across 16 studies, the lack of clarity in addressing investigator influence was a recurring limitation, potentially introducing bias.

### 3.3. Characteristics of the Included Studies

#### 3.3.1. Study Design

Qualitative (60%): Twelve studies used qualitative methods, including semi-structured interviews, focus groups, and narrative analyses to explore SRH barriers and facilitators [3,7,23,25,27,28,29,32,33,34,36,38].

Mixed Methods (40%): Eight studies combined qualitative and quantitative approaches, with three analyzing survey data on SRH access [11,30,35], and five addressing themes like migration, violence, and inequities in SRH [37].

#### 3.3.2. Population Studied by Geographical Area

Studies spanned multiple LAUMIC countries, with findings highlighting unique challenges:

Mexico: Focused on indigenous women, trafficking survivors, and border populations [23,32,34].

Brazil: Addressed Venezuelan migrants and low-income adolescents [24,28,29,39].

Colombia: Explored issues related to forced displacement and rural violence [27,30,31,36].

**Figure 2:**
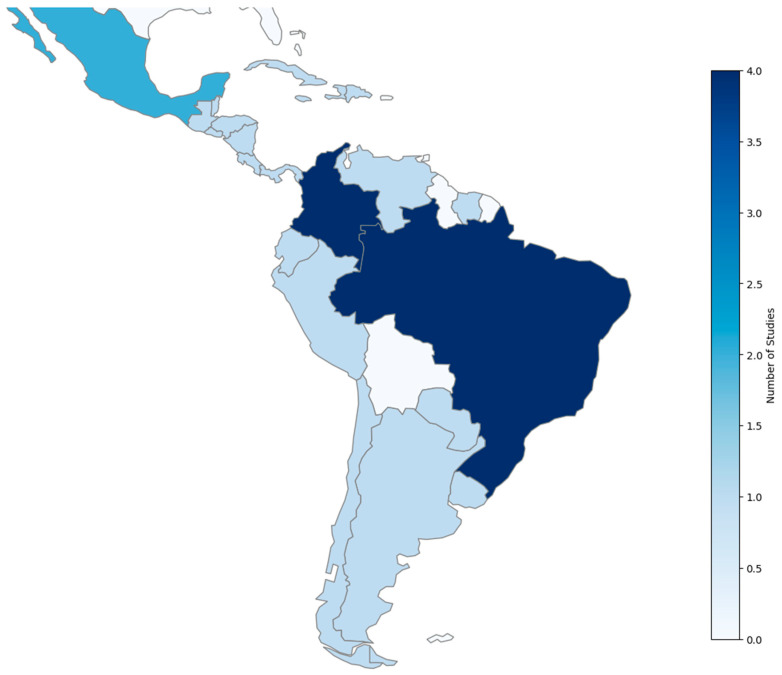
Choropleth map by geography of the included studies. Our own development, the distribution of studies carried out in Latin America on sexual and reproductive health in young women (≤19 years old) is shown. The intensity of the blue colors represents the number of studies conducted in each country, according to the data collected.

Other Countries: Included populations from Guatemala [26], Ecuador [25], Uruguay [7], Chile [33], Peru [11], Argentina [37], and El Salvador [3]. Studies also analyzed cross-border dynamics, such as Mexico–Guatemala [38] and Brazil–French Guiana [35] (see Figure 2).

### 3.4. Findings Synthesized Under the Meta-Aggregation Approach

**Figure 3:**
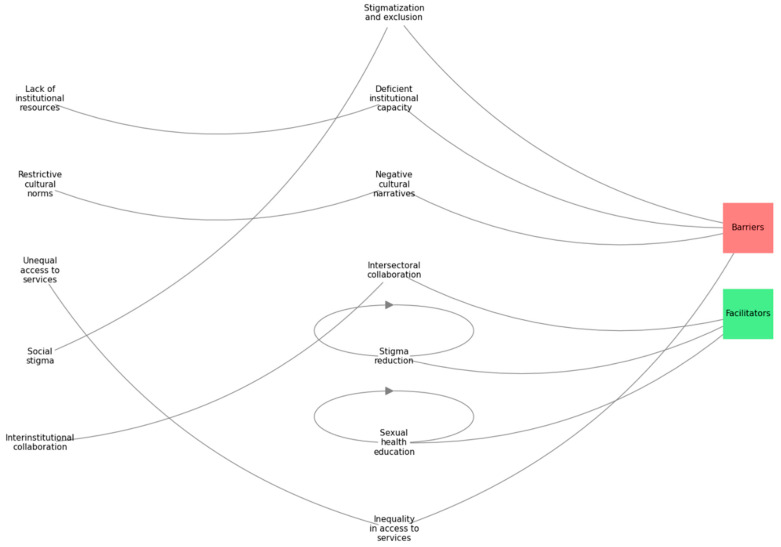
Aggregation diagram phenomenon studied, categories, and meta-themes. Our own development, thematic aggregation, and the relationship between the barriers and facilitators in the context of SRH are illustrated. Meta-themes are grouped into barriers and enablers, presenting the connections of meta-themes to key populations.

The meta-aggregation approach identified three main meta-themes, illustrated in Figure 3, which outlines the relationship between barriers, facilitators, and the key populations studied. Each meta-theme is described in detail to highlight its contribution to understanding SRH access for vulnerable adolescents.

#### 3.4.1. Structural and Contextual Barriers

Structural barriers restrict equitable access to SRH services in vulnerable contexts:

- Inequality in Access: Economic constraints, including insufficient public funding and high service costs, significantly limit SRH access for vulnerable adolescents. Poor healthcare infrastructure and inadequate service coverage further exacerbate these disparities, disproportionately affecting marginalized groups such as ethnic minorities and displaced populations [11,24,28].
- Weak Institutional Capacity: Insufficiently trained healthcare personnel and a lack of culturally sensitive care create systemic barriers to SRH service provision. This weak institutional framework fails to meet the complex needs of vulnerable adolescents, particularly in addressing mental health and SRH simultaneously [25,31,40].
- Stigmatization and Exclusion: Social stigmas surrounding adolescent sexuality, along with institutional discrimination against migrants, sex workers, and unmarried pregnant girls, exacerbate social exclusion and discourage the use of healthcare services. Adolescents often fear judgment from healthcare providers, further limiting their access to essential services [34,38].
- Negative Social Representations: Restrictive societal norms perpetuate gender-based inequities, reinforcing prejudices that hinder reproductive rights and agency for adolescent girls. These cultural constraints not only limit access but also prevent open discussions about SRH needs in many communities [25,30].

#### 3.4.2. Enablers and Strategies to Improve Health Equity

Effective strategies include the following:

- Cross-Sector Collaboration: Collaborative efforts between governmental agencies, NGOs, and local organizations significantly improve SRH outcomes by strengthening healthcare systems, especially in underserved border areas. These partnerships expand service coverage, provide training for healthcare personnel, and ensure resource availability [24,35].
- Sexual Health Education: Comprehensive, culturally tailored sexual education programs enhance adolescent agency by providing accurate information and addressing misconceptions. These programs empower adolescents to make informed decisions, reducing stigma and improving service uptake [28,30].
- Reducing Stigma: Community-driven initiatives play a crucial role in normalizing SRH discussions. Programs led by local leaders and peer educators have successfully reduced stigma around topics like menstrual health and safe abortion, encouraging more adolescents to access services without the fear of judgment [7,38].

#### 3.4.3. Psychosocial Dynamics and Impact on Reproductive Health

Psychosocial factors influence SRH outcomes:

- Impact of Stigma: Stigma associated with abortion and adolescent pregnancy leads to significant psychosocial impacts, including isolation, low self-esteem, and reduced access to healthcare. Criminalization and societal rejection further exacerbate these challenges, particularly for adolescents in rural and marginalized settings [28,34].
- Community Strengthening: Women-led networks, peer support groups, and community alliances have demonstrated effectiveness in mitigating barriers to SRH access. These initiatives foster resilience by providing safe spaces for adolescents to share experiences and access critical resources. They also help challenge stigmatizing norms at the grassroots level, promoting sustainable change in community attitudes [33,39].

## 4. Discussion

We have developed a systematic review with a qualitative synthesis focused on sexual and reproductive health interventions aimed at adolescent women (≤19 years old) living in LAUMICs; we have evaluated barriers and facilitators through a structured thematic analysis. The main findings included the following: (a) Social stigmatization and weak institutional capacity are recognized as structural barriers to SRH access for adolescents in the context of migration, violence, and poverty; (b) cross-sectoral collaboration, community education programs, and (c) proactive interventions to reduce stigma are recognized as facilitators for SRH interventions. Findings on cross-sector collaboration as a key enabler [26,33] are supported by previous studies that highlight the importance of community networks and multisectoral alliances to overcome structural barriers [40,41]. Moreover, the implementation of transnational programs and sexual health education were identified as facilitators in reducing inequities and improving outcomes in cross-border and rural contexts [42].

In this review, the term “social representations” was preferred over “cultural narrative” which often connotes passive storytelling rooted in tradition [43]. Social representations focus on the collective cognitive and social frameworks through which groups construct shared realities. This approach enables a more nuanced understanding of stigma and structural barriers, highlighting how collective beliefs can perpetuate inequality in access to sexual and reproductive health services.

The findings highlight the necessity of adequate funding and robust institutional capacity as fundamental enablers for effectively implementing and sustaining SRH programs. Without these resources, even the most well-designed interventions may fail to achieve equitable outcomes as described in other publications [44].

Identifying structural barriers, such as economic and regulatory constraints, is critical for understanding the multifaceted challenges to sexual and reproductive health (SRH) access. Economic constraints include the high cost of healthcare services and the limited availability of subsidized SRH programs, which disproportionately impact low-income adolescents. Regulatory barriers, such as restrictive policies on contraception or abortion, further limited access and create disparities, particularly for marginalized populations.

Social stigma and cultural factors amplify these challenges, especially in communities where entrenched gender norms dictate limited autonomy for adolescent girls. For instance, unmarried pregnant adolescents often face ostracization, while ethnic minorities may experience discrimination that excludes them from essential SRH services. These barriers not only restrict physical access but also discourage adolescents from seeking care due to the fear of judgment or rejection.

To improve the quality of qualitative research on SRH, it is crucial to proactively address the influence of interviewers and researchers on data collection and interpretation. By acknowledging and minimizing biases, researchers can ensure that the perspectives of vulnerable populations are accurately represented. This includes transparently documenting how the researcher’s positionality and assumptions may shape the findings and providing methodological rigor through reflective practices.

Employing mixed methods (qualitative and quantitative) and adaptive designs, such as snowball sampling through key informants, is vital for effectively studying complex phenomena in hard-to-reach populations and illegal contexts. These methods are particularly relevant in addressing sensitive issues such as human trafficking and clandestine abortions, where traditional data collection approaches may be impractical or unethical. For example, snowball sampling enables researchers to access hidden populations by leveraging trust within communities, while qualitative methods offer rich insights into personal experiences and contextual dynamics [23,28,38].

These methodological approaches are essential for capturing the complex interplay of structural and social barriers, as supported by recent studies emphasizing the value of phenomenological analyses for populations in special situations. Such approaches have uncovered how institutional inadequacies—like insufficiently trained healthcare personnel and a lack of culturally competent services—further compound access issues. For example, in underserved regions, the absence of skilled providers limits the availability of SRH services, creating a cascade of negative outcomes for adolescents.

Community-led initiatives have shown significant promise in addressing these barriers. Programs that normalize SRH services, educate communities about reproductive health, and challenge stigmatizing attitudes at the grassroots level have successfully increased service uptake. Additionally, cross-sectoral collaborations involving governments, NGOs, and local organizations have strengthened institutional capacity, expanded service coverage, and reduced stigma, offering scalable solutions to the structural challenges identified. These findings highlight the importance of tailoring interventions to the unique social and cultural contexts of vulnerable populations, ensuring that solutions are both equitable and sustainable.

Some studies included in this review featured participants over the age of 19; however, they were included because they also encompassed individuals within the target adolescent population and were not exclusive to older age groups. By adopting this inclusive approach, we ensured that critical insights specific to adolescents were retained, allowing for a more comprehensive understanding of the barriers and facilitators impacting adolescent sexual and reproductive health. This strategy ensured that valuable data relevant to the target population were not inadvertently excluded.

Relevance of this study: This review was focused on adolescents from LAUMICs in vulnerable situations in the contexts of migration, violence, and forced displacement, expanding the scope of previous reviews where the focus was exclusively on adolescents who experienced forced displacement [45].

A distinctive aspect of this work was its comprehensive approach, which provides an in-depth characterization of barriers and enablers in SRH. This approach was similar to that described in other reviews, where artificial intelligence tools such as ATLAS.ti were used for the extraction and synthesis of findings [46]; for the present study, we used a step-by-step approach employing templates provided by the JBI methodological manual for qualitative synthesis, which allows the traceability of the sources and results [22].

Contributions to the literature: This study contributes significantly to understanding the exclusion dynamics and facilitators in SRH for adolescents in vulnerable contexts in LAUMICs. This review expands the analysis by including legal barriers, cultural narratives, and the impact of forced migration. The incorporation of underrepresented issues in the literature is highlighted, such as unequal access to services in cross-border areas and the influence of stigma on adolescent sex workers [34,38].

The findings highlight the need to implement cross-sectoral interventions and culturally tailored educational programs to improve access to services, reduce social stigma, and promote equity in SRH.

## 5. Conclusions

This study highlights the significant structural barriers limiting access to sexual and reproductive health (SRH) services for adolescent women (≤19 years) in vulnerable situations in Latin American upper-middle-income countries (LAUMICs). These barriers include entrenched social stigma, negative societal representations, and weak institutional capacities, compounded by economic disparities and restrictive regulatory environments. However, the findings also highlight promising facilitators such as cross-sectoral collaboration, community-led education, and culturally tailored sexual education initiatives that can effectively reduce stigma and enhance service delivery.

To address these challenges, clear and enforceable regulatory frameworks are essential to guarantee free and unrestricted access to SRH services. In addition, integrating human rights education into community and institutional practices can help combat discriminatory attitudes and promote equity. Mixed-method approaches are vital for capturing the nuanced experiences of vulnerable populations, enabling the development of actionable, equitable, and sustainable SRH policies. These findings provide a foundation for evidence-based interventions aimed at addressing the unique needs of adolescents in diverse and marginalized settings.

## Figures and Tables

**Table 1:** Critical analysis of study quality (JBI Critical Appraisal Checklist for Qualitative Research).

Article	Q1	Q2	Q3	Q4	Q5	Q6	Q7	Q8	Q9	Q10	Total, Score	Quality
[3]	Yes	Yes	Yes	Yes	Yes	Yes	Yes	Yes	Yes	Yes	10/10	High
[7]	Yes	Yes	Yes	Yes	Yes	Yes	NC	Yes	Yes	Yes	8/10	High
[11]	Yes	Yes	Yes	Yes	Yes	NA	NC	NA	Yes	Yes	6/10	Moderate
[23]	Yes	Yes	Yes	Yes	Yes	Yes	No	Yes	Yes	Yes	9/10	High
[24]	Yes	Yes	Yes	Yes	Yes	Yes	NC	Yes	Yes	Yes	9/10	High
[25]	Yes	Yes	Yes	Yes	Yes	Yes	NC	Yes	Yes	Yes	9/10	High
[26]	Yes	Yes	Yes	Yes	Yes	Yes	NC	Yes	Yes	Yes	9/10	High
[27]	Yes	Yes	Yes	Yes	Yes	Yes	Yes	Yes	Yes	Yes	10/10	High
[28]	Yes	Yes	Yes	Yes	Yes	Yes	Yes	Yes	Yes	Yes	10/10	High
[29]	Yes	Yes	Yes	Yes	Yes	Yes	Yes	Yes	Yes	Yes	10/10	High
[30]	Yes	Yes	Yes	Yes	Yes	Yes	NC	NA	Yes	Yes	8/10	High
[31]	Yes	Yes	Yes	Yes	Yes	Yes	NC	Yes	Yes	Yes	8/10	High
[32]	Yes	Yes	Yes	Yes	Yes	Yes	NC	Yes	Yes	Yes	8/10	High
[33]	Yes	Yes	Yes	Yes	Yes	Yes	NC	Yes	Yes	Yes	8/10	High
[34]	Yes	Yes	Yes	Yes	Yes	Yes	NC	Yes	Yes	Yes	8/10	High
[35]	Yes	Yes	Yes	Yes	Yes	Yes	NC	NA	Yes	Yes	7/10	Moderate
[36]	Yes	Yes	Yes	Yes	Yes	Yes	NC	Yes	Yes	Yes	8/10	High
[37]	Yes	Yes	Yes	Yes	Yes	No	No	Yes	Yes	Yes	7/10	Moderate
[38]	Yes	Yes	Yes	Yes	Yes	Yes	NC	Yes	Yes	Yes	8/10	High
[39]	Yes	Yes	Yes	Yes	Yes	No	NC	Yes	Yes	Yes	7/10	Moderate

Checklist adapted from the Joanna Briggs Institute (JBI) for critical evaluation [21]. The criteria are defined as follows: Yes—the study meets the criteria; No—the study does not meet the criteria; NA—the criteria are not applicable to the study; NC—the criteria cannot be assessed due to insufficient information. Quality assessment thresholds: High quality—≥8 criteria met; Moderate quality—6–7 criteria met; Low quality—<6 criteria met. Not Applicable (NA): Marked in criterion Q6 for cases where there was no direct contact between researchers and participants [11], and in criterion Q8 for cases where there was a lack of information for evaluating participant voice representation (standardized close-answer surveys [11,30,35].

## Abbreviations

The following abbreviations are used in this manuscript:

**Abbreviation**	**Full Term**
JBI	Joanna Briggs Institute
LAUMICs	Latin American Upper-Middle-Income Countries
LGBTQI+	Lesbian, Gay, Bisexual, Transgender, Queer/Questioning, and Intersex
NGO	Non-Governmental Organization
PRISMA	Preferred Reporting Items for Systematic Reviews and Meta-Analyses
QARI	Qualitative Assessment and Review Instrument
SRH	Sexual and Reproductive Health
UMICs	Upper-Middle-Income Countries

## Appendix A

Table A1 provides supplementary details on the database search strategies employed in this study, offering essential information to ensure transparency and reproducibility without disrupting the flow of the main text. The table outlines the search terms, Boolean operators, filters, and results used across six databases—PubMed, Lilacs, Embase, Dimensions, Scopus, and Google Scholar. These strategies targeted studies focusing on adolescent females (≤19 years) in LAUMICs, examining barriers and facilitators related to contraception, family planning, sexual education, and abortion within the context of ethnic and racial minorities, migration, and forced displacement.

Table A2 details the inclusion and exclusion criteria applied during the selection of studies for the systematic review. It highlights the parameters used to identify relevant research, focusing on adolescent females aged ≤19 years in LAUMICs. The inclusion criteria targeted observational studies addressing the primary and secondary prevention of early pregnancy, particularly in contexts involving ethnic and racial minorities, migration, or forced displacement. Exclusion criteria included studies focusing on populations outside the defined demographic, randomized trials, editorials, or those unrelated to sexual and reproductive health barriers and facilitators. This appendix ensures clarity in the selection process and supports the validity of the review findings.

Table A3 presents the critical appraisal checklist adapted from the JBI Critical Appraisal Checklist for Qualitative Research. This tool was used to systematically evaluate the methodological rigor and relevance of the included studies. The checklist comprises 10 key questions addressing congruence between methodology and data collection, researcher positioning, the representation of participants’ voices, and ethical adherence. This structured evaluation ensures the inclusion of high-quality research, providing a robust foundation for the synthesis of findings.

Table A4 provides a detailed summary of excluded studies and reasons for their exclusion, complementing the information in Table A1, Table A2, Table A3 and Table A4.

**Table A1:** Database Search Strategies.

Source	Search Strategy	Results
PubMed	(((teenage OR adolescent) AND (female OR girl OR women)) AND (Argentina OR Brazil OR Colombia OR “Costa Rica” OR Cuba OR Dominican OR Ecuador OR Salvador OR Guatemala OR Mexico OR Paraguay OR Peru)) AND (contracept* OR “family planning” OR “birth control” OR “pregnancy interruption” OR (education AND (sexual OR sex)) OR (abortion AND (induced OR criminal))) AND (“Ethnic and Racial Minorities”[Mesh] OR “Human Migration”[Mesh] OR “Armed Conflicts”[Mesh]) Filters: English, Portuguese, Spanish, from 2019–2023	
Lilacs	(((teenage OR adolescent) AND (female OR girl OR women)) (Argentina OR Brazil OR Colombia OR “Costa Rica” OR Cuba OR Dominican OR Ecuador OR Salvador OR Guatemala OR Mexico OR Paraguay OR Peru)) AND (contracept* OR “family planning” OR “birth control” OR “pregnancy interruption” OR (education AND (sexual OR sex)) OR (abortion AND (induced OR criminal))) AND ((minorities AND (ethnic OR racial)) OR (migration OR “Force displacement” OR refugees) OR “Armed Conflicts”) AND la:(“en” OR “es” OR “pt”) AND (year_cluster: [2019 TO 2023]) AND instance:”regional”	
Embase	(((teenage OR adolescent) AND (female OR girl OR women)) AND (Argentina OR Brazil OR Colombia OR “Costa Rica” OR Cuba OR Dominican OR Ecuador OR Salvador OR Guatemala OR Mexico OR Paraguay OR Peru)) AND (contracept* OR “family planning” OR “birth control” OR “pregnancy interruption” OR (education AND (sexual OR sex)) OR (abortion AND (induced OR criminal))) AND (’ethnic group’/exp OR ’migration’/exp OR ’war’/exp) AND (2019:py OR 2020:py OR 2021:py OR 2022:py OR 2023:py) AND ([english]/lim OR [portuguese]/lim OR [spanish]/lim)	24
Dimensions	(((teenage OR adolescent) AND (female OR girl OR women)) AND (Argentina OR Brazil OR Colombia OR “Costa Rica” OR Cuba OR Dominican OR Ecuador OR Salvador OR Guatemala OR Mexico OR Paraguay OR Peru)) AND (contracept* OR “family planning” OR “birth control” OR “pregnancy interruption” OR (education AND (sexual OR sex)) OR (abortion AND (induced OR criminal))) AND (mesh_terms:(“Ethnic and Racial Minorities”) OR mesh_terms:(“Human Migration” OR mesh_terms:(“Armed Conflicts”)) Filters: Publication type: “Article”, from 2019 to 2023. Search in title and abstract	
Scopus	((teenage OR adolescent) AND (female OR girl OR women)) AND (argentina OR brazil OR colombia OR “Costa Rica” OR cuba OR dominican OR ecuador OR salvador OR guatemala OR mexico OR paraguay OR peru))) AND (contracept* OR “family planning” OR “birth control” OR “pregnancy interruption” OR (education AND (sexual OR sex)) OR (abortion AND (induced OR criminal))) AND (& ethnic AND group’/exp OR & migration’/exp OR’; war’/exp) AND PUBYEAR > 2018 AND PUBYEAR < 2024	
Scholar google	(((teenage OR adolescent) AND (female OR girl OR women)) (Argentina OR Brazil OR Colombia OR “Costa Rica” OR Cuba OR Dominican OR Ecuador OR Salvador OR Guatemala OR Mexico OR Paraguay OR Peru)) AND (contracept* OR “family planning” OR “birth control” OR “pregnancy interruption” OR (education AND (sexual OR sex)) OR (abortion AND (induced OR criminal))) AND ((minorities AND (ethnic OR racial)) OR (migration OR “Force displacement” OR refugees) OR “Armed Conflicts”) Filters: Publication type: “Review Article”, from 2019 to2023	

This appendix serves as a detailed reference for replicating the search and understanding how relevant studies were identified for the systematic review. *: is used as a truncation symbol in the search query (e.g., contracept*), it indicates that the search includes all possible variations of the root word. For example: contracept* retrieves contraception, contraceptive, contraceptives, etc.

**Table A2:** Inclusion and Exclusion Criteria.

Criterion	Inclusion	Exclusion
Population	Studies or data collected in the territory of the countries defined by LAUMCs. This ensures the relevance of the research context. Studies that include women aged ≤ 19.	Wrong population: Studies focusing on men, transgender, or LGBTIQ populations, or excluding women aged ≤ 19.
Phenomenon of Interest	Observational studies (cohorts, case-controls, systematic reviews, case series) on the primary and secondary prevention of early pregnancy (focusing on family planning, termination of pregnancy, or sexuality education).	Wrong outcome: Studies with endpoints irrelevant to early pregnancy prevention or SRH. Wrong publication type: Editorials, expert opinions, letters, or other non-research articles.
Context	Studies focused on ethnic or racial minority settings, refugees, and victims of displacement or armed conflict.	Wrong setting: Studies conducted outside the defined LAUMC scope or not focused on vulnerable populations. Background article: Studies providing general information not directly contributing to the research objectives.

This appendix outlines the criteria used to determine the eligibility of studies for inclusion in the systematic review, ensuring relevance to the research objectives.

**Table A3:** Checklist for Critical Appraisal in JBI Qualitative Research.

Question	Yes	No	Not Clear	Not Applicable
1. Is there congruence between the stated philosophical perspective and the research methodology?	☐	☐	☐	☐
2. Is there congruence between the methodology, questions, and objectives of the research?	☐	☐	☐	☐
3. Is there congruence between the research methodology and the methods used to collect data?	☐	☐	☐	☐
4. Is there congruence between the research methodology and the representation and analysis of the data?	☐	☐	☐	☐
5. Is there congruence between the research methodology and the interpretation of the results?	☐	☐	☐	☐
6. Does the researcher situate himself culturally or theoretically in the study?	☐	☐	☐	☐
7. Is the influence of the researcher on research and vice versa addressed?	☐	☐	☐	☐
8. Are the participants and their voices adequately represented?	☐	☐	☐	☐
9. Does the research meet current ethical criteria or, in the case of recent studies, is there evidence of ethical approval by an appropriate body?	☐	☐	☐	☐
10. Do the conclusions presented in the research report derive from the analysis or interpretation of the data?	☐	☐	☐	☐

Adapted from the JBI Critical Appraisal Checklist for Qualitative Research [21]. This appendix provides the adapted checklist used to critically appraise the qualitative studies included in the review. It ensures a structured evaluation of each study’s methodology, data analysis, and ethical adherence.

**Table A4:** Extracting Data from Individual Studies (QARI Tool).

Author, Year	Methodology	Method	Phenomenon of Interest	Geography	Characteristics of the Participants	Authors’ Conclusions
De Jesus, 2019 [3]	This qualitative study employed an emic approach, focusing on the internal perspectives, beliefs, and experiences of young people in the Northern Triangle (the tri-border region of El Salvador, Guatemala, and Honduras). The study explored their experiences and perceptions within this context. The sample included young people aged 15 to 24 years, residing in urban areas, with at least one close family member who had immigrated to the U.S. Participants were selected through snowball sampling, facilitated by key community informants. A total of 60 in-depth interviews and six focus groups (two per country) were conducted in Spanish within private community spaces. The findings were synthesized using thematic analysis, facilitated by ATLAS.ti software.	Qualitative	Impact of generalized violence on the health and human security of young people in the Northern Triangle, identifying protective and resilience factors.	Urban context in Guatemala, El Salvador, and Honduras, regions with high rates of violence.	Young people aged 15–24, living in urban areas and with at least one close family member who immigrated to the U.S. Note: Although the interviews were not specifically focused on sexual and reproductive health, they included the target population within the context of violence, highlighting its relevance in the design of sexual and reproductive health policies for adolescents	Widespread violence in the “Northern Triangle” of Central America poses a systemic threat to the health and well-being of young people, affecting them at the individual, family, and community levels. Despite this context, young people show resilience through their commitment to education, the support of local and transnational family networks, and their participation in community youth groups. However, state responses, such as “iron fist” policies, have been ineffective and have exacerbated the problem. The authors advocate adopting the Latin American Collective Social Medicine approach to address the structural roots of violence and propose multisectoral strategies that promote health, sustainable development, and equity in the region.
Ituarte, 2021 [7]	A cross-sectional, exploratory, and descriptive study was carried out; the sample included 14 female adolescents between 17 and 19 years old, residing in Montevideo, Uruguay; the participants were selected through intentional sampling in public health services. Data was collected through semi-directed face-to-face interviews in four cases and by telephone in ten cases. The synthesis of results was carried out by content analysis, using inductive coding to identify emerging themes and patterns in the participants’ experiences.	Qualitative	Experiences of adolescents when deciding to terminate a pregnancy in a legal framework.	Montevideo, Uruguay.	Adolescent women between 17 and 19 years of age after the voluntary termination of pregnancy.	The decisions were influenced by socially accepted reasons, such as educational continuity and a lack of emotional and economic preparation. Mothers played an important role as support and guidance figures. The legal framework of abortion was perceived positively, providing security and legitimacy to the process. Social stigmas persist that affect the subjective experience of abortion.
Mendoza, 2019 [11]	A secondary data analysis was carried out from the Survey on the Living Conditions of the Venezuelan Population in Peru (ENPOVE) and the Flow Monitoring of the Venezuelan Population administered by IOM and UNICEF. The sample included Venezuelan migrants residing in Peru between 2017 and 2019, with national representation. Living conditions and access to health services were reported among Venezuelan migrants and the Peruvian urban population.	Mixed Qualitative–Quantitative	Impacts of Venezuelan immigration on public health in Peru and the specific needs of the migrant population.	Main cities of Peru, with emphasis on Lima and Callao.	The study included migrants from Venezuela (of Venezuelan, Peruvian, or other origins) across all age groups. The 18 to 44-year-old group was specifically included and accounted for three-quarters of the total population..	There are significant barriers to access to health services, particularly in sexual and reproductive health. Immigration represents an opportunity to strengthen the health system, taking advantage of the potential of Venezuelan professionals and technicians. Challenges such as the integration of health services, universal insurance, and the reduction in inequalities persist.
Acharya, 2019 [23]	Exploratory study using snowball sampling technique. Indigenous women victims of human trafficking were identified and interviewed in Monterrey, Mexico. For identification, key informants were selected to identify the victims, and semi-structured and in-depth interviews were subsequently conducted with 68 women, of whom 46 were victims of labor trafficking and 22 of sex trafficking. The interviews were conducted between February 2016 and March 2017. The synthesis of findings combined a quantitative approach, using frequencies, with a critical analysis of the discourse under the narratives and contexts of exploitation experienced by the participants.	Qualitative	Sociodemographic characteristics of indigenous women victims of labor and sex trafficking.	Monterrey, Mexico, including its 12 municipalities, with a focus on urban and exploitative contexts.	Indigenous women victims of labor and sex trafficking between the ages of 12 and 45 (44% ≤ 20 years). Predominantly from the states of Veracruz, Oaxaca, San Luis Potosí, and Puebla.	Human trafficking is a multidimensional phenomenon that particularly affects indigenous women due to economic, social, and family factors. Victims suffer multiple forms of violence (physical, sexual, psychological) and severe injuries, such as skin damage, fractures, and hearing and visual impairments. Exploitation in informal sectors and the lack of mental and physical health services perpetuate the cycle of vulnerability. National programs for the care of victims of trafficking in Mexico need to incorporate a comprehensive approach that addresses the physical and mental needs of victims.
Bahamondes, 2020 [24]	Cross-sectional study to evaluate access to sexual and reproductive health (SRH) services among Venezuelan migrant women in Brazil. Face-to-face interviews were conducted with 405 Venezuelan women between 18 and 49 years old living in Boa Vista and Pacaraima, Roraima, Brazil. The questionnaire included open and closed questions, adapted from the Minimum Initial Services Package for the Brazilian context. Non-Spanish-speaking indigenous women and adolescents < 18 years of age were excluded. The synthesis of results included a descriptive analysis as well as multivariate analyses to identify variables associated with a lack of access to SRH services.	Mixed Qualitative–Quantitative	Sexual and reproductive health (SRH) needs of Venezuelan migrant women, including access to services, contraception, and prenatal and postnatal care.	Boa Vista and Pacaraima, in the state of Roraima, Brazil.	Venezuelan migrant women from 18 to 49 years old. Residents of UNHCR shelters or informal settlements in Boa Vista and Pacaraima.	There are significant gaps in the provision of SRH services for this population, especially in family planning and antenatal/postnatal care. The Brazilian health system faces significant limitations in capacity and resources to meet the needs of the migrant population. A coordinated approach between the government sectors, academia, and international organizations is needed to improve access and quality of services.
Boira, 2021 [25]	Mixed analysis (quantitative and qualitative) to examine gender-based violence in Ecuador. The quantitative analysis was based on data from the Attorney General’s Office of Ecuador (2018), disaggregating the reports of crimes of sexual violence by province. The qualitative analysis focused on interviews and focus groups with participants of various professions, ethnicities, and ages, providing a multidimensional perspective of the phenomenon in the province of Imbabura, Ecuador. The interviews and focus groups included professionals involved in the management of cases of gender-based violence.	Mixed Qualitative–Quantitative	Sexual violence against women, with emphasis on rural and indigenous areas of Imbabura, Ecuador.	Imbabura Province, Ecuador, located in the Andean region.	Indigenous and mestizo women from rural communities. Professionals in the field of justice, health, and social work. Variety in gender, age, and professional roles in focus groups.	Crimes of sexual violence, including abuse and rape, have increased significantly in Ecuador in recent years, particularly in Imbabura. Patriarchal culture plays a critical role in perpetuating sexual violence. Victims face substantial barriers to reporting, such as lack of institutional support, stigmatization, and the normalization of violence. There is an urgent need to raise awareness of sexual violence and strengthen comprehensive sex education to address its structural causes. Impunity remains a major issue, reinforced by traditional and weak justice systems. A multidimensional approach, incorporating qualitative analyses, is recommended to better understand the phenomenon.
Boyce, 2020 [26]	Mixed design, developed between 2012 and 2013 in three urban centers of Guatemala: Guatemala City, Escuintla, and San Marcos. Data collection was conducted using census sampling and modified time–place sampling, including 1216 adult female sex workers who had exchanged sex for money in the past 12 months. Computer and audio-assisted interviews (ACASI) were used to collect sensitive data, supplemented by biological tests to detect HIV and other sexually transmitted infections (STDs). This approach allowed detailed information to be obtained on both the sociodemographic factors and the health aspects of this population.	Mixed Qualitative–Quantitative	Assess how early initiation in the sex trade affects HIV risk and other vulnerabilities (violence, lack of HIV education, non-use of condoms).	Three urban areas in Guatemala: Guatemala City, Escuintla, and San Marcos.	Women ≥ 18 years of age; sex workers, including internal and foreign migrants, with different levels of education.	Sex workers who started before the age of 16 are at an increased risk of being HIV-positive, not receiving HIV education, and experiencing violence to force sex work. The interaction between migration and age of onset significantly influences health risks. Primary prevention needs to be strengthened to prevent adolescents from entering the sex trade and reduce HIV prevalence in these vulnerable populations.
Cadena-Camargo, 2020 [27]	This ethnographic study explored the experiences of adolescent pregnancy in the context of forced displacement. Data collection included 20 in-depth interviews to collect the life stories of displaced women in the community of Ciudad Bolívar, Bogotá. The data were analyzed through a thematic analysis using qualitative coding tools (Nvivo), organizing the information into emerging themes that describe the experiences and challenges of adolescents in this context of vulnerability.	Qualitative	Experiences of adolescent pregnancy among internally displaced women in Bogotá, including their meanings and challenges in the context of armed conflict and displacement.	Ciudad Bolívar, Bogotá, Colombia, a town characterized by high levels of violence and poverty, recipient of a displaced population.	Women victims of forced displacement who experienced teenage pregnancy. Note: The participants’ current age ranged from 18 to 35 years. However, since the history of first motherhood from the age of 14 was an inclusion criterion, the findings of this study are relevant to the characterization and intervention strategies for adolescent pregnancy.	Adolescent pregnancy among displaced women is closely linked to rural violence and traumatic childhood experiences. While these women perceive motherhood as a challenge, they also view it as an opportunity to build a family and find purpose in their lives. However, dynamic displacement and gender inequalities further exacerbate their vulnerabilities, particularly due to the lack of social and economic support. A policy and healthcare approach is needed that goes beyond biomedical prevention and contraceptive methods, incorporating the perspectives and lived experiences of affected women.
Ferrari, 2020 [28]	A qualitative study using in-depth semi-structured interviews explored the experiences of adolescents who underwent clandestine abortions in a favela in the South Zone of Rio de Janeiro. The participants were adolescents aged 15 to 17 years, who had undergone the procedure between the ages of 12 and 17. They were selected through snowball sampling, with the support of key informant networks. Data collection followed interview guides adapted from the GRAVAD study, covering topics such as sexual initiation, contraceptive use, abortion itineraries, and decision-making processes. The findings were synthesized using thematic and categorical content analyses.	Qualitative	Itineraries, decisions, and risks associated with clandestine abortion among adolescents.	Favela of the South Zone, Rio de Janeiro, Brazil.	Average age: 16 years. Self-reported race/ethnicity: 9 out of 10 identified as black or brown.	Abortion pathways reflect deep social, economic, and gender vulnerabilities. Loneliness and family/maternal exclusion are central to the experience. It is urgent to recognize abortion in adolescents as a legitimate phenomenon of sexual and reproductive health.
Ferreira, 2020 [29]	In-depth episodic interviews were conducted for eight women aged 19 to 28 years, recruited through informal networks in the metropolitan region of Recife, Brazil. The findings were synthesized using thematic coding based on content analysis, identifying patterns and dynamics related to the intersections of gender, class, and race in the participants’ experiences with voluntary pregnancy termination.	Qualitative	Analysis of how class and race shape the abortive itineraries of young women.	Recife Metropolitan Region, Brazil.	Women aged 19 to 28 years who faced decisions related to the termination of pregnancy.	Young white and middle-class women have shorter itineraries, with stronger support networks and more conflicting decisions. Young black and working-class women narrate longer itineraries, with more precarious support networks and immediate decisions. The itineraries are crossed by fear, support, relief, loneliness, and violence.
González-Hernández, 2020 [30]	Cross-sectional research using the CUES (Condom Use Errors/Problems Survey) instrument adapted for men and women. We included 775 adults aged 18 to 26 years (53.4% female and 46.5% male) who required to have used at least three condoms in the three months before the study and to complete all questions on the CUES instrument. Participants were recruited via Facebook and completed an online survey. In addition to the CUES, scales such as the Sexual Opinion Survey, the Sexual Assertiveness Scale, and the Multidimensional Condom Attitudes Scale were used. Descriptive measures and tests of association between variables related to errors and problems in condom use were reported.	Quantitative	Phenomenon of Interest: Errors and challenges in condom use among young adults, analyzed through the lens of gender, sexual orientation, and relationship stability.	Participants from different regions of Colombia.	Colombian young adults, between 18 and 26 years old.	The most prevalent mistakes and problems include not checking the condom for visible damage and using it after initiating penetration. Problems associated with condom-related erectile dysfunction (CAEP) were reported at higher rates than in North American studies (47.4% during application and 36% during use). Attitudes toward condoms, sexual assertiveness, and partner context all influence the likelihood of mistakes/problems.
Hudcovská, 2020 [31]	Semi-structured interviews were conducted with 42 professionals selected through purposive sampling, and 165 internal psychosocial health reports of adolescents (57 women and 108 men) were analyzed. The results were synthesized using an inductive content approach to identify the main themes and sub-themes emerging in the narratives and records analyzed.	Mixed Qualitative–Quantitative	Psychosocial conditions of adolescents disassociated from illegal armed groups in Colombia.	Cities of Colombia: Bogotá, Medellín, Cali, Villavicencio, and Armenia.	Professionals interviewed: related to the care of victims of the armed conflict (psychologists, social workers, educators, and coordinators). Reports analyzed from the following adolescents who were attended in specialized psychiatric/psychological consultation.	Adolescents face significant impacts (e.g., sexual abuse, and mental health problems). Challenges in the program include administrative barriers, lack of health insurance portability, and high staff turnover. It is recommended to incorporate alternative strategies such as artistic activities and active listening to strengthen psychosocial accompaniment.
Küng, 2021 [32]	A total of 34 interviews were conducted with obstetricians/gynecologists and general practitioners (17 objectors and 17 non-objectors) and 12 focus groups involving 82 participants, including physicians, nurses, social workers, and administrative staff. Data were collected in two states of Mexico (Jalisco and State of Mexico) and in four departments of Bolivia (La Paz, Chuquisaca, Potosí, and Santa Cruz). Semi-structured guidelines adapted by the type of participant (objectors or non-objectors) were used. The results were synthesized using a thematic approach with inductive coding, using ATLAS.ti software to organize and analyze the findings.	Qualitative	Use and abuse of conscientious objection (CO) as a barrier to access to legal abortion.	Jalisco and Estado de México (Mexico); La Paz, Chuquisaca, Potosí, and Santa Cruz (Bolivia).	The majority of participants were women (76% in FGDs, 56% in IDIs). The sample included a diverse range of roles, such as doctors, nurses, psychologists, social workers, and administrative staff. Note: Although the interviewed population was older than 19 years (beyond the primary age group of interest in this review), the insights from sexual and reproductive health professionals provide a valuable perspective for improving programs related to voluntary pregnancy termination.	The primary reasons for denying abortion services include lack of knowledge about laws and protocols, fear of legal repercussions, value judgments toward patients, and advanced pregnancy. Additionally, administrative barriers, stigmatization, and inadequate education on abortion-related laws further exacerbate the issue. To address these challenges, the authors propose enhancing the training of both medical and administrative staff and establishing clear protocols to prevent the misuse of conscientious objection (CO).
Larrea, 2022 [33]	A feminist epistemological study was carried out based on approaches to health inequalities. Eleven semi-structured interviews were conducted with a population selected by convenience sampling, and the population included women who used the Women Help Women (WHW) service between November 2018 and April 2019, in Santiago, Chile. Decisions about abortion, experiences with the use of misoprostol, and perceptions about the quality of care received were explored. The results were synthesized by thematic coding, using the triangulation of researchers to ensure the validity and depth of the findings.	Qualitative	Evaluation of the quality of care provided by WHW in the context of abortion self-management.	Santiago, Chile.	The study included women aged 23 to 36 years, most of whom had university education and formal employment. Only two participants were mothers at the time of the interview. Note: Although the study population was older than 19 years (beyond the primary age group of interest in this review), the findings on opportunities for program improvement can be extrapolated to early pregnancy cases in the context of self-managed abortion.	The experience of the self-management of abortion was marked by stigma, fear, and privacy. WHW was rated positively for its speed, emotional support, and accessibility, although some participants noted limitations in instant communication and professional care.
Maldonado, 2020 [34]	Ethnographic research with a focus on narratives and lived experiences extracted by participant observation, in-depth interviews, and informal conversations. The sample included cisgender women sex workers, aged between 17 and 50 years, from Mexico and Central America (Honduras, El Salvador, and Guatemala), recruited in sex workspaces in the Soconusco–Chiapas region. The results were synthesized through a thematic analysis based on inductive coding to identify patterns and meanings in the collected narratives.	Qualitative	Impacts of the anti-trafficking device on the lives of migrant sex workers on the Mexico–Guatemala border.	Soconusco Region, Chiapas, Mexico.	Cisgender women, mostly migrants and of Central American origin. Occupations include self-employed sex work and roles in establishments linked to the sex trade (waitresses, “ficheras”).	The anti-trafficking mechanism criminalizes sex work and irregular migration, exacerbating state and structural violence. Anti-trafficking policies are marked by notions of gender and class that infantilize women and perpetuate racial and gender stereotypes. Women sex workers are not simply victims; they are agents with capacities for resistance and political agency.
Mosnier, 2019 [35]	Cross-sectional descriptive research based on the KAP model (knowledge, attitudes and practices). The sample included 621 individuals, aged 18 years, ≥ Oiapoque (Brazil) and Saint-Georges-de-l’Oyapock (French Guiana). A random sampling was used for selected households in streets previously drawn in both countries; A structured questionnaire of 77 questions was administered through individual interviews conducted by trained local mediators. Descriptive statistics and bivariate analyses were reported to identify associations between the variables of knowledge, attitudes, and practices with the object of study.	Quantitative	Levels of knowledge about HIV, risky practices, and stigmatization in border populations.	Border area between Brazil (Oiapoque) and French Guiana (Saint-Georges-de-l’Oyapock).	Female predominance (60.9%). Native languages: Portuguese (77%) and Creole or Amerindian languages (15.3%). Educational level: 56.4% with secondary or higher education. Precarious socioeconomic situation, with only 16.4% in formal paid employment.	Knowledge about HIV was deficient in 45.9% of the participants; myths such as mosquito transmission were common. Risk behaviors (20.5%) were more frequent in men and younger people. There were high levels of stigmatization (74.8%), higher among speakers of Creole and Amerindian languages. Recommendations include community communication strategies in native languages and increased cross-border collaboration on prevention and treatment.
Palacios—Perdomo, 2021 [36]	Exploratory research with a phenomenological approach. The sample consisted of 37 participants distributed in three focus groups and 12 semi-structured interviews. Focus groups included social leaders, young people with adolescent pregnancy, and young people with experiences close to adolescent pregnancy, recruited in the rural areas of Palmira and El Cerrito, in the Amaime River basin, Colombia. Open-ended question guides designed to address the social determination of health and the concepts of structural, cultural, and direct violence according to Galtung and Fischer were used. A thematic analysis was applied with the codification of emerging subcategories, organized into general, specific, and singular domains to delve into the social dynamics and violence associated with adolescent pregnancy.	Qualitative	Social and contextual determinants of adolescent pregnancy in rural areas affected by the armed conflict in Colombia.	Amaime River Basin Region (Palmira and El Cerrito), Colombia.	Young people aged 14 to 18 who experienced teenage pregnancy, social leaders, and health personnel. Participants lived in rural areas with high geographical dispersion and marked by historical violence.	Adolescent pregnancy is influenced by structural conditions such as poverty, gender inequities, and cultural roles that assign motherhood as a destiny. There are forms of cultural, symbolic, and structural violence that perpetuate inequalities. Peacebuilding requires a multisectoral approach to address social determinants, strengthen the community fabric, and promote gender equality.
Ramón, 2020 [37]	A mixed study approach was used, which included the review of jurisprudence and literature, followed by a cross-sectional survey and semi-structured interviews. In the survey, 269 sexual and reproductive health professionals in Argentina participated, and the interviews included 11 managers and heads of reproductive health departments; the participants were recruited through convenience sampling through pro-choice networks. Online questionnaires for the survey and semi-structured interviews were used to collect qualitative information. The results were synthesized under an initial coding process and thematic analysis, guaranteeing consistency and depth in the findings.	Mixed Qualitative–Quantitative	To examine the uses and consequences of conscientious objection (CO) in the provision of legal abortions in Argentina.	Argentina, with emphasis on the public health system.	Most participants were aged 30 to 39 years (39%), with general practitioners and gynecologists being the most represented specialties. A total of 88% provided sexual and reproductive health services, and 74% performed legal abortions. Additionally, 45% of participants reported having no religious affiliation. Note: Although the interviewed population was older than 19 years (beyond the primary age group of interest in this review), the insights from sexual and reproductive health professionals offer a valuable perspective for improving programs related to voluntary pregnancy termination.	Conscientious Objection (CO) is exercised beyond religious or moral motives, often influenced by political, social, or personal convenience. Its main consequences include delays in access to services, tensions within healthcare teams, and the stigmatization of reproductive health services. To address these issues, the authors propose a regulatory framework that limits CO to explicitly religious or moral reasons, while excluding its institutional application and preventing its use by individuals in leadership roles.
Rocha-Jiménez, 2020 [38]	An ethnographic approach was conducted. The study included 25 female migrant sex workers from Guatemala, Honduras, El Salvador, and Nicaragua, selected through purposive sampling. The inclusion criteria were to be a migrant, ≥18 years old, and to perform sex work in the border region. In-depth semi-structured interviews and participant observation were used in bars, nightclubs, and streets. Data were analyzed using thematic coding and an inductive approach to identify patterns related to substance use practices and risks associated with HIV/STIs.	Qualitative	Impact of migration on substance use practices and HIV/STI risks among women sex workers.	Border region between Mexico and Guatemala, specifically in Tapachula, Chiapas.	Women ≥ 18 years old. Nationalities: Guatemala, Honduras, El Salvador, and Nicaragua. High levels of socioeconomic vulnerability and limited access to health services.	Migration increases exposure to risks related to the use of unsafe substances and sexual practices. Women reported an increased use of alcohol and drugs to cope with stress and precarious working conditions. Structural barriers such as discrimination and lack of access to health services increase vulnerability to HIV/STIs. Interventions adapted to cross-border and culturally sensitive contexts are needed.
Soeiro, 2021 [39]	A cross-sectional study was conducted using self-administered questionnaires in Spanish, based on the “Menstrual Practice Needs Scale” (MPNS-36). In total, 167 adolescent and young Venezuelan migrant women, aged between 12 and 24 years, were invited to participate; 142 completed the questionnaire. Recruitment took place in informal settlements in Boa Vista, Brazil. A structured questionnaire with open-ended and closed-ended questions was used, designed to collect information on menstrual hygiene practices, access to hygiene kits, and sanitation conditions. Descriptive results were presented for categorical variables and means for continuous variables. The study was approved by the Ethics Committee of the University of Campinas and an informed consent process adapted for unaccompanied minors was implemented.	Mixed Qualitative–Quantitative	To identify menstrual poverty problems in Venezuelan migrant adolescent women and young women.	Boa Vista, Roraima State, Brazil.	Average age: 17.7 years. Mainly shopkeepers behind the Boa Vista bus station. In total, 71% migrated due to lack of economic opportunities. In total, 46% did not receive hygiene kits since their arrival.	There is a clear neglect of the menstrual hygiene needs of Venezuelan migrant adolescents and young women. The main barriers include a lack of menstrual products, unsafe bathrooms, and poor sanitary conditions. They propose collaboration between NGOs, international agencies, and local governments to guarantee menstrual kits and menstrual dignity.

Adapted from QARI. Available online: https://jbi-global-wiki.refined.site/space/MANUAL/355861446/Appendix+3.3:+JBI+Qualitative+data+extraction+tool (accessed on 8 November 2024).
